# Synthesis and Characterization of Fiber-Reinforced Resorcinol Epoxy Acrylate Applied to Stereolithography 3D Printing

**DOI:** 10.1021/acsomega.1c04566

**Published:** 2021-11-11

**Authors:** Prashil. Dharmesh. Desai, Ramanand. Namdev. Jagtap

**Affiliations:** Department of Polymer Engineering & Surface Coating Technology, Institute of Chemical Technology, Mumbai 400 019, India

## Abstract

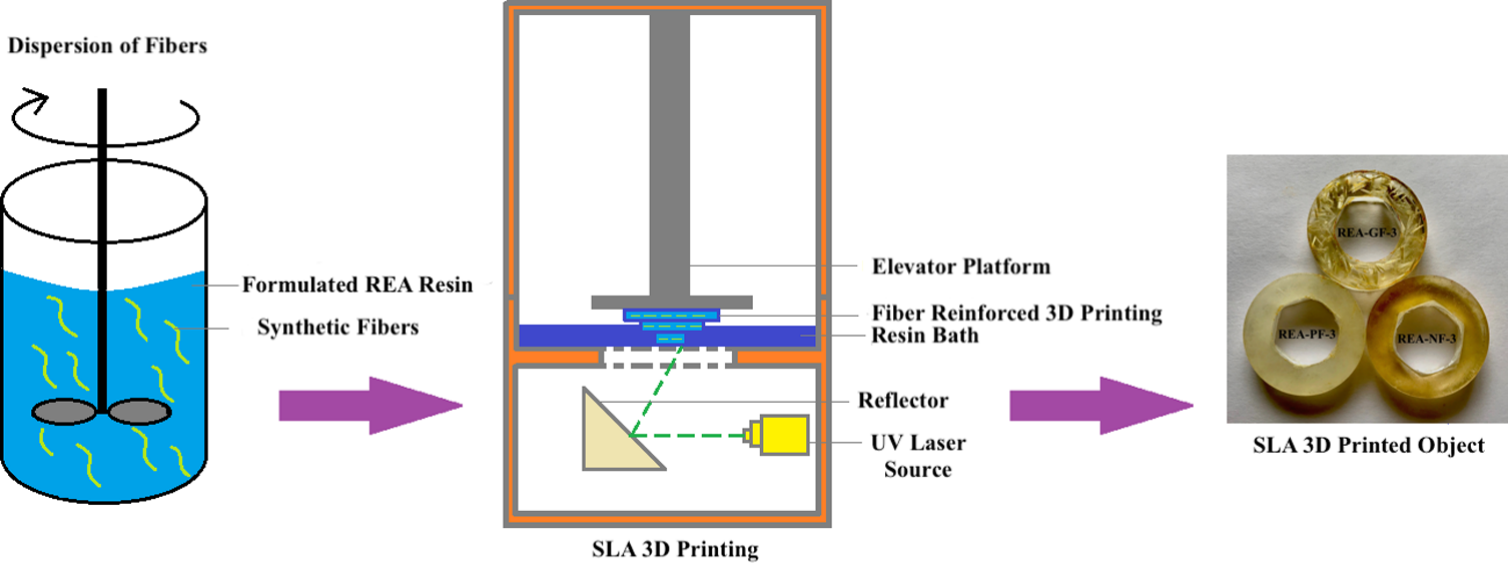

As perceived, fiber-reinforced photocuring resins possess various distinctive advantages over traditional reinforced resin systems. This includes a rapid curing rate, energy efficiency, no volatile organic compounds, high strength, good thermal stability, and chemical resistance. Printing 3D composite objects using vat polymerization techniques has been a center of interest where photocuring resins are applied. In our present study, we have synthesized resorcinol-based diglycidyl ether, that is, a resorcinol-based epoxy resin, and further acrylated to the resorcinol epoxy acrylate oligomer. This oligomer was further formulated to photocuring resins using a suitable quantity of reactive diluents and photoinitiators. Now, three types of synthetic fibers, that is, glass fibers, nylon fibers, and polyester fibers, were incorporated in this formulated resin at different loading percentages. The oligomer synthesized was analyzed for structural conformation using Fourier transform infrared spectroscopy and ^13^C nuclear magnetic resonance. Further, we comparatively examined the rheological behavior of prepared formulations, and compatible formulations were applied to stereolithography 3D printers. Finally, physical, mechanical, thermal, transmittance, and morphological characteristics were comparatively analyzed for prepared UV-cured composites. The outcomes obtained during characterizations of UV-cured composites will be inevitably reflected in 3D-printed objects.

## Introduction

Resorcinol chemistry has seen an enormous leap in the field of polymer and coating technology. Resins such as epoxy, aromatic polyesters, polyamides, and formaldehyde based on resorcinol and its derivatives are commercially manufactured polymers. These resorcinol-based polymers have established a diverse profile for their applications as adhesives, coatings, plastic moldings, rubber composites, etc.^1,2^ The reported studies reveal that the resorcinol-based epoxy acrylate resin exhibits the quality of a high-performance material. The amount of unsaturation, that is, acrylate functional groups, is much higher in the resorcinol epoxy acrylate (REA) oligomer when compared to conventional bisphenol A epoxy acrylate, which will successively lead to optimum cross-linking at the curing stage.^3^ It also exhibits low viscosity, a fast curing rate, high strength, and excellent thermal stability.^4^ Moreover, it also has excellent wetting and dispersing properties, which aids in uniform blending for pigments, fillers, and additives.^1,2^ Due to the numerous advantages attributed to the REA oligomer, it was considered suitable to formulate as a photocuring resin for stereolithography (SLA) 3D printing.

Fiber-reinforced polymer composites are used enormously in construction, automobiles, aeronautics, defense, marine industries, etc. The reinforcement improves strength, hardness, thermodynamic properties, and chemical resistance.^5−7^ Traditionally, fiber reinforcement was introduced in polymers such as polyethylene terephthalate, polypropylene, high-density polyethylene, polycarbonate, and polyamide.^8^ Later, development of reinforced resin demonstrated improvement in resin bond strength, flexural strength, load-bearing capacity, fracture strength, and fatigue resistance. Commonly, epoxy and unsaturated polyester resins are used in the manufacture of fiber-reinforced polymers.^9^ Fibers are usually reinforced during polymer processing or formulating resin matrixes, which form reinforced composites while cross-linking. Some significant reasons for reinforcing synthetic fibers over natural fibers are no pretreatment required, dimensional stability, uniform orientation, low weathering, less moisture absorption, easy availability, and cost-effectiveness.^10^ The compatibility of reinforced fibers within the polymer matrix depends on many factors such as the material of construction, surface treatment, orientation, dimension stability, intermolecular bonds, chemical interactions, and so forth.^11^

Immense progression has been observed in the technology of photocuring resins over other conventional resin curing systems. Single-component formulation, a fast curing rate, environment friendliness, and time and energy efficiency are some of the befitting advantages provided by the photocuring resin technology.^12−14^ Studies are reported using nanofiller reinforcement such as silicon dioxide, graphene oxide, alumina, titanium carbide, and carbon black in photocuring resins for additive manufacturing.^15−17^ Experiments are demonstrated based on the reinforcement of natural and synthetic fibers in photocuring resins to create 3D-printed fiber-reinforced composites.^18^ There are numerous advantages in terms of strength, thermal stability, chemical resistance, abrasion, etc. Alongside benefits, some limitations were observed, such as porosity, air bubble formation, limited reinforcing materials, an increase in viscosity, and filler sedimentation while making reinforced composites using photocuring resins.^19^ Thus, to solve the above downsides, an appropriate quantity of reactive diluents is complemented while dispersing fillers in photocurable resins, which aids reduction in resin viscosity, increases dispersion stability, and enhances the cross-linking in a resin matrix. The study reported the use of next-generation biocompatible nanofibers for making a 3D object using the additive manufacturing technology. These printed composites can be high-performance materials for dental applications, bone transplants, prosthetics, etc.^20^ With the advancement of the SLA 3D printing technology and the chemistry of photocuring resins, these reinforced resins have perceived a new perspective for commercial applications.

**Figure 1:**
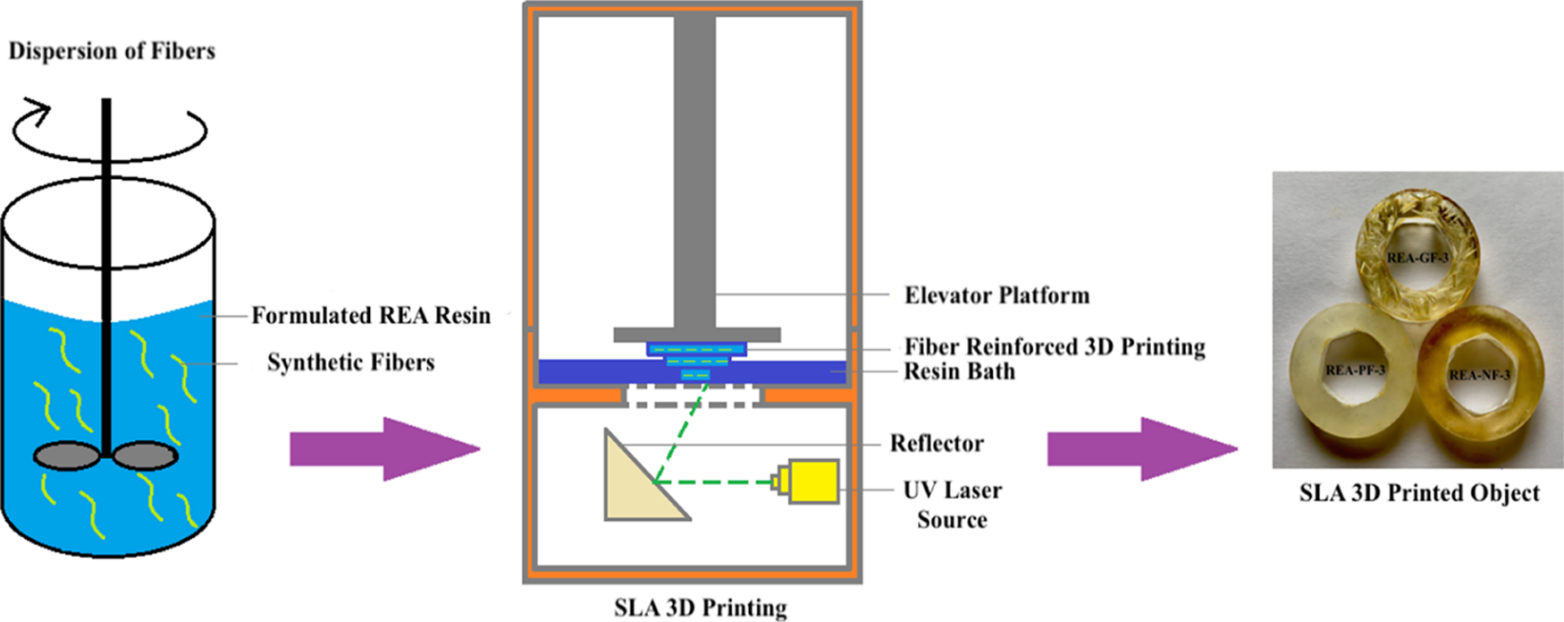
Fiber-reinforced 3D printing using the SLA technology.

In a new attempt, we have synthesized a low-viscous REA oligomer from resorcinol-based diglycidyl ether. As demonstrated in Figure 1, the resin formulation compatible with the SLA 3D printer was developed by blending a suitable quantity of reactive diluents and photoinitiators. Now, three different types of synthetic fibers, glass fibers, nylon fibers, and polyester fibers (GF, NF, and PF), are dispersed separately in concentrations of 1, 3, and 5% in the formulated REA resin. The REA oligomer synthesized was characterized for its structural conformation and purity, followed by a rheological analysis of the fiber-reinforced formulated resin. A comparative study on physicochemical properties, optical transmittance, mechanical performance, thermal stability, and high-resolution microscopy was performed for prepared UV-cured composites. Further, we selected 3% fiber-reinforced formulations for crafting objects using an SLA 3D printer. Additionally, we analyzed the printed objects based on visual examination for their printing resolution, accuracy, and orientation of fibers.

## Experimental Section

### Materials

Resorcinol (Atul Ltd.), epichlorohydrin (Atul Ltd.), ethanol (Merk), acrylic acid (Merck), trimethylolpropane triacrylate (TMPTA, Merk), hexanediol diacrylate (HDDA, Merk), tetra-*n*-butyl ammonium bromide (TBAB, Merk), dichloro methane (MDC, Merk), phenylbis (2,4,6-trimethylbenzoyl) phosphine oxide (Irgacure 819, Ciba), 1-hydroxycyclohexyl-phenyl ketone (Irgacure 184, Ciba), hydroquinone (HQ, Merk), butylated hydroxytoluene (BHT, Merk), GF (KDM Chemicals), NF (GSFC), and PF (Reliance Industries Ltd.) with dimensions of a 6 mm length and a 1.5 Denier thickness.

## Method

### Synthesis of Resorcinol Diglycidyl Ethers

**Scheme 1:**
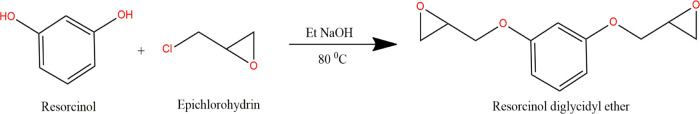
Synthesis Process of RDGE

The resin was synthesized in a 2000 mL round-bottom reactor equipped with a reflux condenser, Dean and Stark, a stirrer, a thermometer, and a nitrogen inlet. Resorcinol (173.79 g, 1.57 mol) reacted with epichlorohydrin (1168.35 g, 12.62 mol) in the presence of ethanolic NaOH (157.85 g, 3.94 mol of NaOH in 795 g, 1000 mL of ethanol) added dropwise at 80 °C with continuous removal of ethanol from another side to maintain the reaction volume. The reaction was carried out under reflux conditions for 2 h, and the water of reaction generated was distilled off. In the end, the reaction mass was filtered out hot to remove the salt and washed with distilled water. Further, it was vacuum-distilled to remove residual water along with an excess of reactants and solvents. Here, the product obtained is resorcinol diglycidyl ether (RDGE), as represented in Scheme 1.^21^

### Synthesis of the UV-Curable REA Oligomer

**Scheme 2:**
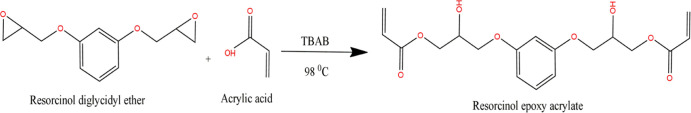
Synthesis Process of the REA Oligomer

The oligomer was synthesized in a 500 mL round-bottom reactor, equipped with a reflux condenser, stirrer, thermometer, and nitrogen inlet. RDGE (130.52 g, 0.58 mol) synthesized above was reacted with acrylic acid (169.47 g, 2.35 mol) in the presence of the base catalyst TBAB (1 wt %), the solvent MDC (10 wt %), the inhibitor HQ (0.1 wt %), and the antioxidant BHT (0.1 wt %). The reaction temperature was slowly increased to 90 °C and maintained for 18 h. At the end of the reaction, the product was washed with alkali and brine solution to remove excess acrylic acid. Vacuum distillation was carried out to remove traces of solvents and moisture. Hence, the final product obtained is the REA oligomer as represented in Scheme 2.^22,23^

### Formulating the UV-Curable Resin

The REA oligomer synthesized was formulated with an appropriate quantity of the reactive diluents (10% w/w) TMPTA and (10% w/w) HDDA, and the photoinitiators (0.5% w/w) irgacure 819 and (0.5% w/w) irgacure 184 are also blended in equal parts. The further formulated resin is reinforced with different percentages of synthetic fibers under high-speed stirring, as represented in Table 1.

**Table 1:** Formulating the REA Resin with Variation in Fiber Types and Percentage Loading

resin formulation (wt %)	REA oligomer	TMPTA	HDDA	irgacure 819	irgacure 184	GF	NF	PF
REA 0	79.2	9.9	9.9	0.5	0.5			
REA-GF 1	78.4	9.8	9.8	0.5	0.5	1		
REA-GF 3	76.8	9.6	9.6	0.5	0.5	3		
REA-GF 5	75.2	9.4	9.4	0.5	0.5	5		
REA-NF 1	78.4	9.8	9.8	0.5	0.5		1	
REA-NF 3	76.8	9.6	9.6	0.5	0.5		3	
REA-NF 5	75.2	9.4	9.4	0.5	0.5		5	
REA-PF 1	78.4	9.8	9.8	0.5	0.5			1
REA-PF 3	76.8	9.6	9.6	0.5	0.5			3
REA-PF 5	75.2	9.4	9.4	0.5	0.5			5

### Silicone-Molded Die-Cut Dumbbell Shape Composites

**Figure 2:**
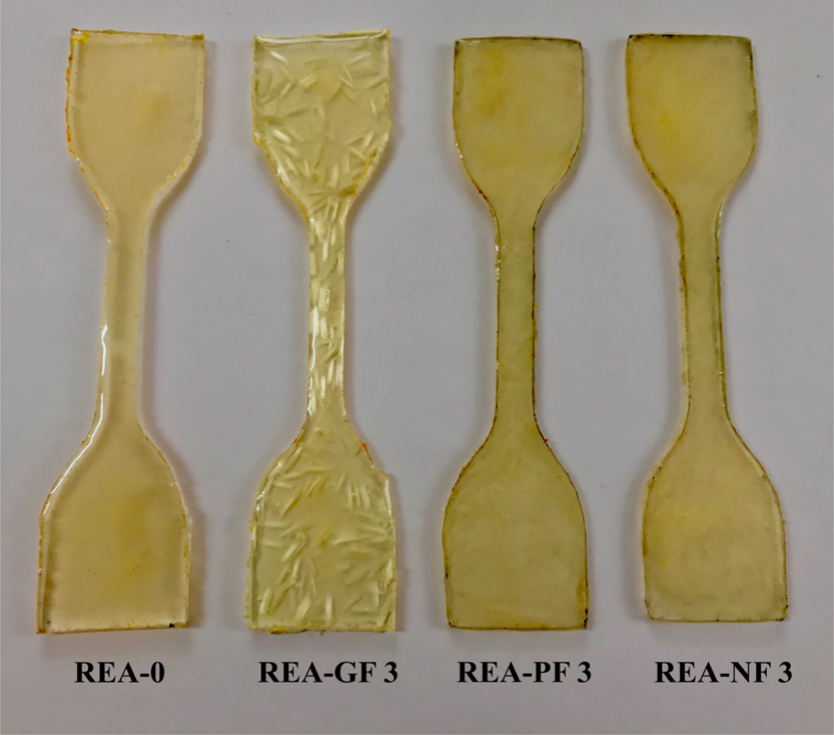
UV lamp-cured dumbbell shape specimens of REA-0 and 3% fiber-reinforced REA formulation, that is, REA-GF 3, REA-PF 3, and REA-PF 3. Similar specimens were prepared using 1 and 5% fiber-reinforced REA formulations from Table 1.

All the REA formulations were cast in silicone molds (150 × 25 × 2 mm^3^) and then irradiated under a UV curing machine (UNIQUE UV curing machine, India) with a high-pressure mercury lamp (wavelength: 405 nm, power: 4 kW) for 5 s. For post-processing requirements, the cured specimens from these molds were washed with isopropyl alcohol (IPA) and exposed to UV light (wavelength: 365 nm, power: 36 W) for 10 min to ensure complete curing. The resin in a silicone mold was photocured using a UV lamp that emulates the wavelength of the UV laser built-in SLA 3D printer. Additionally, post-processing conditions were parallelly followed in both the cases, thus reducing the deviation in cross-linking density of cured specimens obtained from both the processes. The specimens are further molded to a dumbbell shape with the help of a die cut (ASTM D638), as shown in Figure 2. All the UV-cured dumbbell shape specimens prepared from the above formulation were subjected to high-end characterization.

### SLA 3D Printing

**Figure 3:**
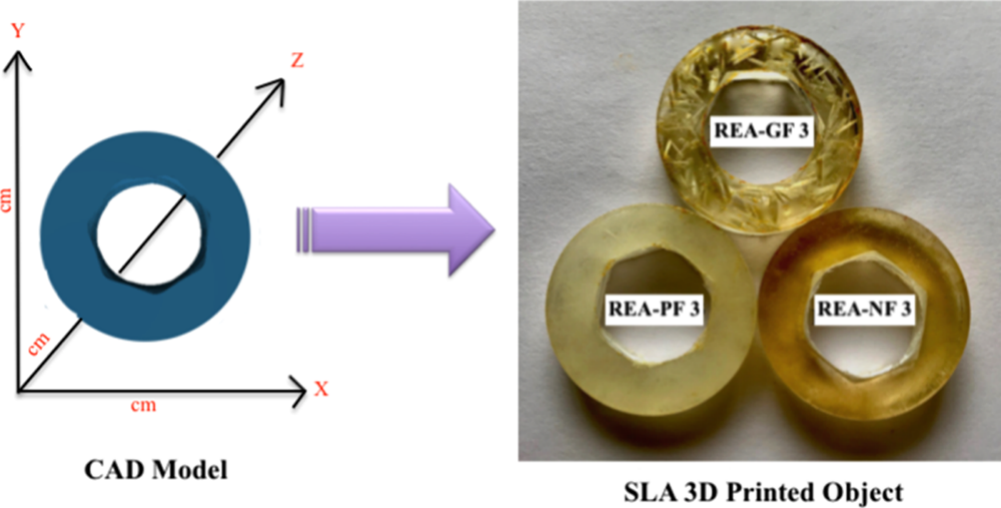
SLA 3D-printed fiber-reinforced composites. The composites are crafted using 3% fiber-reinforced REA formulation, that is, REA-GF 3, REA-PF 3, and REA-PF 3.

Formlabs 2 was used as a desktop SLA 3D printer to craft 3D objects. This printer uses a 250 mW UV laser beam having an intensity of a 405 nm wavelength and a buildup volume of 145 × 145 × 175 mm^3^. 3D objects were printed using a raw SLT file downloaded from the open source and uploaded to Formlabs 2 software. The Z-resolution, that is, the thickness was set to 100 μm, which will increase the printing layer thickness to the maximum. This change improves the processability of the fiber-incorporated resin and the uniform dispersion of fibers among all horizontal printed layers. The SLA 3D printer was operated in the open mode to regulate the printing speed and intensity of the UV laser as we use the lab-formulated photocuring resin. Studying the rheological characteristics and physicochemical properties for all the REA formulations, the 3% fiber-reinforced REA formulation was considered for 3D printing. The REA-GF 3, REA-NF 3, and REA-PF 3 formulations were subsequently poured into the resin bath, and 3D composite objects were crafted from it, as shown in Figure 3. Composites were later subjected to post-processing requirements. To ensure complete curing, they were washed with IPA and exposed to UV light (wavelength: 365 nm, power: 36 W) for 10 min to ensure complete curing.

## Characterization

### Fourier Transform Infrared Analysis

Fourier transform infrared (FTIR) spectroscopy was performed on synthesized RDGE and its acrylated resin (uncured and cured). Spectra were recorded using a Bruker ALPHA II equipped with platinum attenuated total reflection (ATR) accessory featuring monolithic diamond crystals, along with the analysis range of 4000–400 cm^–1^ and a resolution of 4 cm^–1^.

### ^13^C Nuclear Magnetic Resonance Analysis

^13^C Nuclear magnetic resonance (^13^C NMR) spectroscopy was performed on the synthesized REA oligomer using a Bruker Advance 400 Hz in the chemical shift range of 0–240 ppm with the CDCl_3_ solvent. After the acylation of epoxide groups, sharp and broad resonating peaks of carbon atoms were examined to imitate the backbone structure of the synthesized resin.

### Viscosity Analysis

An Anton Paar MCR 102 rheometer attached with a parallel-plate geometry was used to measure the viscosity of fiber-reinforced formulated photocuring resins at 25 °C. The comparative study for resin viscosity was made based on the types of fibers used and the percentage of fibers incorporated in resins. The diameter of the geometry was 50 mm, and the gap between the two geometries was 1 mm.

### Hardness

The Sore D hardness test was performed following ASTM D2240 on fiber-reinforced UV-cured composites. A comparative study was established to analyze the deviation in surface hardness alongside the type and amount of fibers used as a reinforced material in the formulated REA photocuring resin.

### Gel Content

The gel content was estimated for the UV-cured composites by immersing a known weight of the sample (*W*_1_) in the tetrahydrofuran (THF) solvent. After 24 h, the sample was removed and oven-dried to measure the final weight (*W*_2_). The gel content was measured using eq 1
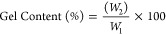
1where (*W*_1_) = weight of the sample before immersion and (*W*_2_) = weight of the sample after immersion and drying.

### Water Absorption

The water absorption percentage was evaluated according to ASTM D-570 for UV-cured REA composites. The composites were dried in an oven until a constant weight was achieved (*W*_i_) and then dipped in water for 24 h, and the final weight was taken (*W*_f_). The difference in weight measures water absorbance following eq 2
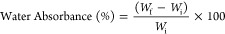
2

### Chemical Resistance

The chemical resistance of UV-cured composites of formulated REA was examined following ASTM D543. The composites were separately dipped in HCL (10% w/w) and NaOH (10% w/w) solution for 24 h at room temperature. Later, the composites were observed for any surface defects and deformation at the end of the test.

### Mechanical Analysis

A universal testing machine (UTM) Instron 3365 evaluated the tensile properties with a load cell of 1 kN and a deformation speed of 2 mm/min. The above-prepared UV-cured dumbbell-shaped specimens were tested for tensile strength and percentage elongation, and a comparative study was generated.

### Optical Analysis

The optical properties of different fiber-reinforced UV-cured composites were studied using ultraviolet–visible (UV–vis) spectroscopy (Agilent Cary 60). Transmittance properties are measured and comparatively evaluated within the range of 400–800 nm.

### Differential Scanning Calorimetry

The glass-transition temperature was comparatively analyzed for the UV-cured REA formulations using differential scanning calorimetry (DSC). A Mettler Toledo DSC 3 was used to scan the cured samples in the temperature range of 25–400 °C at a heating rate of 20 °C min^–1^ with constant nitrogen flow. The samplings are weighed under 10 mg and placed in a suitably sealed aluminum crucible.

### Thermogravimetric Analysis

The degradation behavior of different fiber-reinforced UV-cured composites was studied using a Mettler Toledo TGA 2. The temperature range was set to 25–600 °C with a heating rate of 10 °C min^–1^ while performing thermogravimetric analyses (TGAs). The degradation temperatures were comparatively evaluated for reinforced composites at 10, 50, and 100% degradation alongside the percentage char yield at the maximum temperature.

### High-Resolution Microscopic Analysis of the UV-Cured Composite

The surface morphology of fiber-reinforced UV-cured composites was studied using a high-resolution microscopy image. The images of UV-cured composites were captured at fracture points using an Olympus DSX 1000 digital microscope in the range of 2θ = 20–60^0^.

## Results and Discussion

### Fourier Transform Infrared Analysis

**Figure 4:**
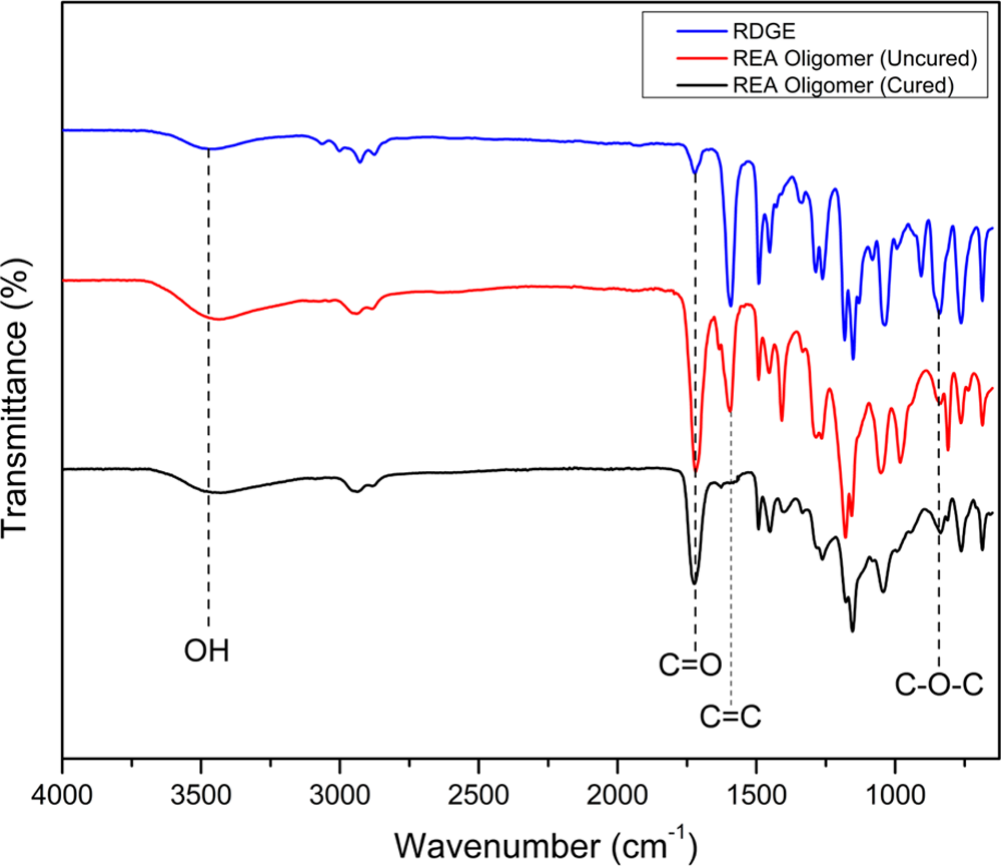
FTIR spectra of RDGE and the REA oligomer (uncured and cured).

Comparative analysis was performed on FTIR spectra, as shown in Figure 4, for the synthesized RDGE, REA oligomer (uncured), and UV-cured REA. The first synthesis of RDGE shows a C–O–C (oxiran group) peak at 915 cm^–1^. Further, the REA oligomer is synthesized, which shows characteristic peaks of OH, C=O, and C=C at 3450, 1729, and 1635 cm^–1^, respectively, whereas the above oxirane group peak at 915 cm^–1^ got consumed. Finally, after UV-curing the REA oligomer, it was seen that C=C and C=O band stretching reduced to a considerable extent; this confirms the cross-linking of the resin. Thus, group determination for synthesized resins and their cured product was confirmed using the FTIR test.

### ^13^C Nuclear Magnetic Resonance Analysis

**Figure 5:**
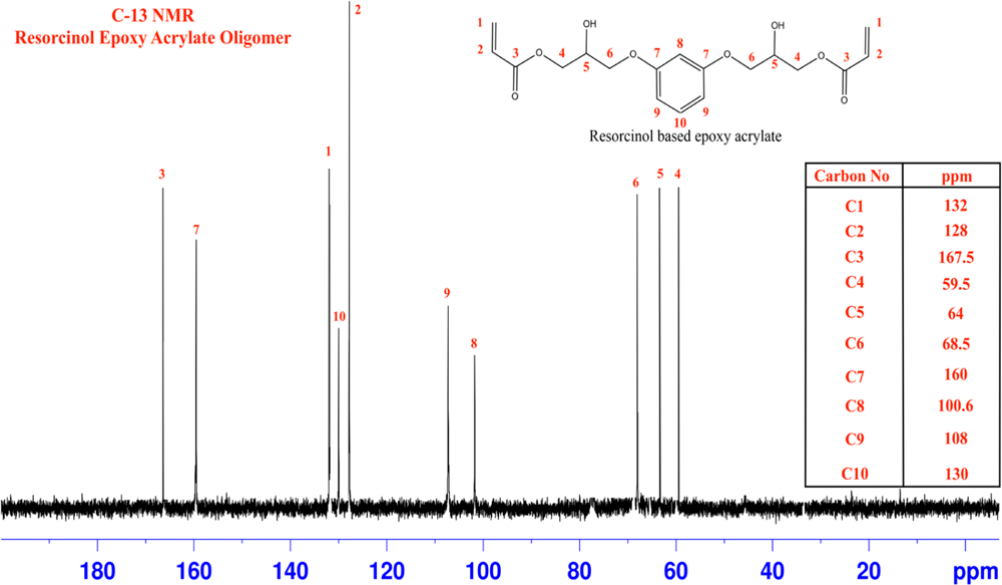
^13^C NMR spectra of the synthesized REA oligomer.

The structural characterization was performed on synthesized REA oligomers using ^13^C NMR spectroscopy. This characterization helps in structural confirmation by showing resonation peaks for the respective carbon atoms and evaluating the product’s purity. As represented in Figure 5, ^13^C NMR spectra show that the resonation peaks at δ 132 ppm (C1) and δ 128 ppm (C2) signify acryloyl double bonds, whereas absorbance at δ 167.5 ppm (C3) represents carbonyl carbon of ester. There is absorption at the δ 59.5 ppm (C4) peak due to the deshielded effect of the carbonyl group. Similarly, peak absorption was noticed at δ 64 ppm (C5) following the deshielding effect of the adjacent hydroxyl group attached to the carbon. The observed absorbance peaks at δ 68.5 ppm (C6) and δ 160 ppm (C7) are a result of oxygen linkage with carbon, that is, the formation of the ether group, thus confirming that the high-purity REA oligomer is synthesized with no or minimal byproduct in it.

### Viscosity Analysis

**Figure 6:**
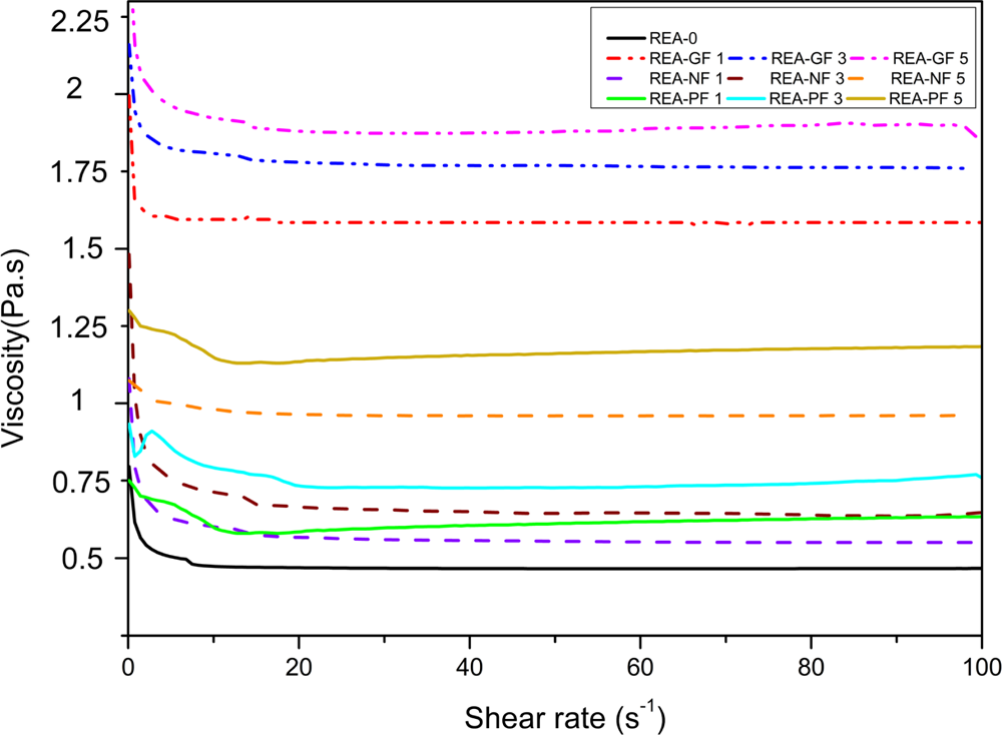
Rheological behavior of fiber-reinforced formulated resins under increasing shear rate.

Figure 6 demonstrates the viscosity of the reinforced formulated resin with the increase in shear rate. The amount of rising viscosity of formulated reinforced resins is mainly dependent on the density of the synthetic fibers. The densities of GF, NF, and PF are 2.44, 1.15, and 1.38, respectively; hence, their effect is subsequently reflected in the viscosity of the reinforced formulated resin. The viscosities obtained after 1% fiber reinforcement in the formulation of REA-GF 1, GRE-NF 1, and REA-PF 1 are 1.58, 0.55, and 0.625 Pa s, respectively. Furthermore, with the increase in the quantity of GF, NF, and PF reinforcement, there was a corresponding rise in the viscosity of the formulated REA resin. At the time of 3D printing, it was apparent that low-viscous resin formulations in the range of 0.5–7.3 Pa s would provide the best processability in SLA printing of 3D objects. Hence, 3% fiber-loaded formulations, that is, REA-GF 3, GRE-NF 3, and REA-PF 3, having viscosities of 1.76, 0.64, and 0.73 Pa s, respectively, were selected for SLA 3D printing.

### Characterization of UV-Cured Composites

#### Hardness, Gel Content, Water Absorption, and Chemical Resistance

As represented in Table 2, the hardness, gel content, water absorption, and chemical resistance were comparatively evaluated for all the UV-cured composites.

**Table 2:** Hardness, Gel Content, Water Absorption, and Chemical Resistance for UV-Cured Composites

resin formulation	fiber amount (%)	shore D hardness	gel content (%)	water absorption (%)	chemical resistance
REA 0	0	82	99.2	0.76	pass
REA-GF 1	1	85	99.4	0.68	
REA-GF 3	2	89	99.6	0.55	pass
REA-GF 5	3	91	99.7	0.51	
REA-NF 1	1	83	98.1	0.88	
REA-NF 3	2	87	98.7	0.92	pass
REA-NF 5	3	89	99.1	1.33	
REA-PF 1	1	80	98.3	0.72	
REA-PF 3	2	85	98.9	0.65	pass
REA-PF 5	3	86	99.2	0.61	

The hardness of fiber-reinforced composites mainly depends on the type and amount of fiber reinforced. The outcomes from comparative evaluation of UV-cured fiber-reinforced composites indicated that GF composites provide maximum shore hardness, followed by NF and PF composites, respectively. GF possesses the highest strength and stiffness among all the reinforced fibers and develops exemplary embedment in the cured resin, which leads to superior hardness of the composite. Furthermore, the hardness of the material also increases with the rise in percentage reinforcement of fibers. The hardness results have a significant influence on the tensile strength of the composite.

The gel contents for all the UV-cured composites were sufficiently high, which were above 98%. Among all the synthetic fibers used, GF-reinforced composites deliver the highest gel content, whereas we obtained nearby results in the cases of NF and PF. GF’s high resistance to the solvent and the substantial binding property in the resin matrix while curing lead to this outcome. Further, NF offers good resistance to the solvent, followed by PF. Hence, these properties are subsequently reflected in their respective composites.

The water absorption test indicates that UV-cured GF-reinforced composites hold the lowest percentage of water, whereas UV-cured NF-reinforced composites retain the highest percentage of water. Due to the high hygroscopic nature of NF, the water absorption percentage of NF composites was found to be maximum, followed by PF and GF composites.

After immersing UV-cured composites in acid and alkali solutions for 24 h, the composites underwent a visual examination. All UV-cured composites successfully passed the chemical resistance test, and there were no signs of blistering effects or softening materials on the composites. The high cross-linking nature and good hydrogen bonding in epoxy acrylate develop superior resistance to acid and alkali media. Additionally, the reinforced synthetic fibers also possess excellent chemical resistance and thus uphold composite properties while testing.

### Mechanical Analysis

**Figure 7:**
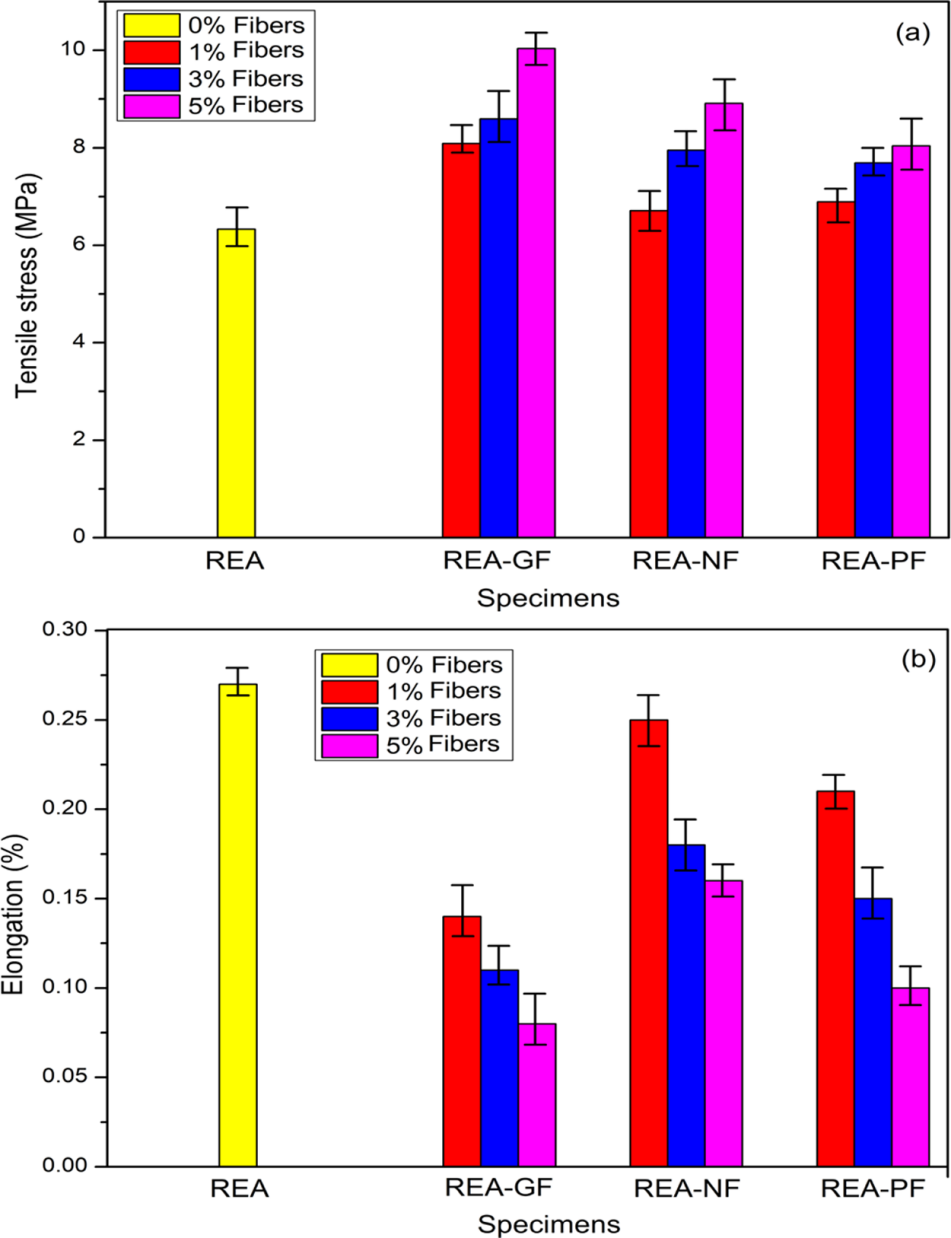
(a) Tensile stress and (b) % elongation of fiber-reinforced UV-cured composites having different concentrations of the fiber reinforced.

The tensile stress and percentage elongation were comparatively evaluated for UV-cured specimens using a universal tensile machine. The non-reinforced UV-cured specimen, that is, REA-0, exhibited a tensile strength of 6.33 MPa alongside a percentage elongation of 0.27%. The tensile graphs in Figure 7a demonstrate that tensile stress escalates sharply with increased percentage reinforcement of fibers. However, percentage elongation tends to reduce with the increase in the concentration of fibers for all UV-cured composites, as represented in Figure 7b. Reinforcing 5% GF generates the highest tensile strength of 10.04 M Pa with the lowest percentage elongation of 0.08. The appropriate embedment and orientation of GF alongside its self-strength in cured composites lead to this behavior.^24^ The reinforcement of 5% NF and 5% PF yields the tensile strengths of 9.81 and 8.04 M Pa, respectively, while the percentage elongation was 0.16 and 0.1, respectively. The NF-reinforced composite exhibited high elongation, followed by PF and GF. This elongation property in the UV-cured composite is primarily due to the characteristic behavior of the synthetic fiber reinforced.^25^ The 3D-printed composited object will reflect similar tensile properties as found in the above composites.

### Optical Analysis

**Figure 8:**
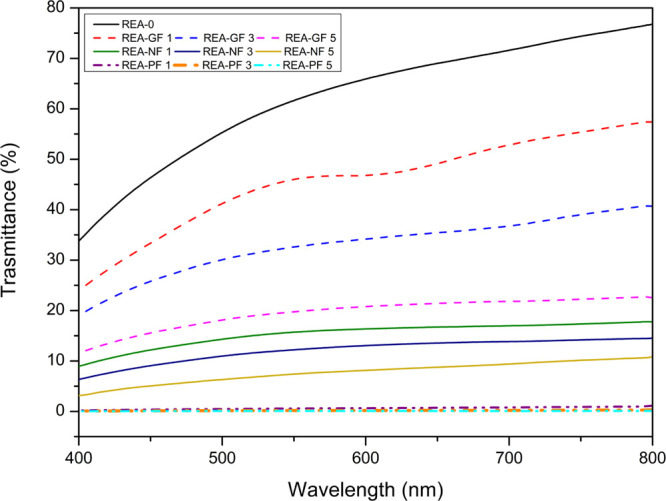
Comparative study for transmittance (%) emitted by UV-cured composites.

Optical properties were evaluated for all the UV-cured composites using the UV–vis spectroscopy test, which shows percentage transmittance in the range of 400–800 nm. The transmittance graph exhibited in Figure 8 reveals that UV-cured REA-0 gives the highest % transmittance. It also illustrates that percentage transmittance decreases in UV-cured composites with the increase in fiber reinforcement. The transmittance of fiber-reinforced composites mainly depends on the properties of fibers, such as the material of construction, refractive index, geometry, orientation, and dispersion.^26^ Generally, transmittance increases by using fibers having a lower refractive index alongside appropriate loading and achieving uniform distribution of fibers in cured composites. The refractive indices of GF, NF, and PF used in this experiment are 1.54, 1.59, and 1.73, respectively.^27,28^ Further analysis of the graph shows that incorporating GF gives maximum transmittance, whereas the lowest was in the case of PF. The transmittance results obtained for all the UV-cured composites comply with the refractive index of incorporated fibers. Thus, the refractive index of fibers will significantly impact the transmittance property of the UV-cured composites. Visually seeing UV-cured composites confirms that transmittance results are quite relatable.

### Differential Scanning Calorimetry

**Figure 9:**
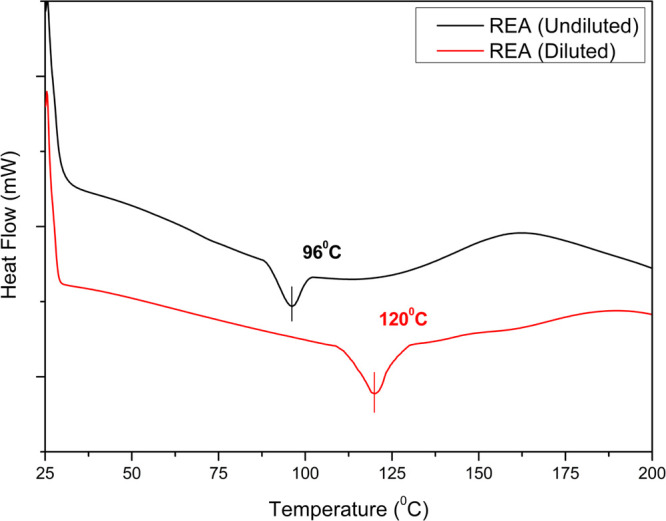
Comparison of the glass-transition temperature of UV-cured REA, undiluted and diluted.

The comparative evaluation was performed for glass-transition temperatures between UV-cured undiluted REA and diluted REA, that is, REA-0 using DSC. The DSC graph in Figure 9 shows that *T*g of UV-cured undiluted REA and diluted REA was 96 and 120 °C, respectively. The rise in *T*g was mainly due to the blending of reactive diluents that increases the unsaturation and thus enhances the cross-linking density while curing. This improved cross-linking will promote good intermolecular adhesion, which ultimately leads to a rise in *T*g. Hence, the diluted REA formulation will provide better thermal stability in UV-cured composites at higher temperatures.

### Thermogravimetric Analysis

**Figure 10:**
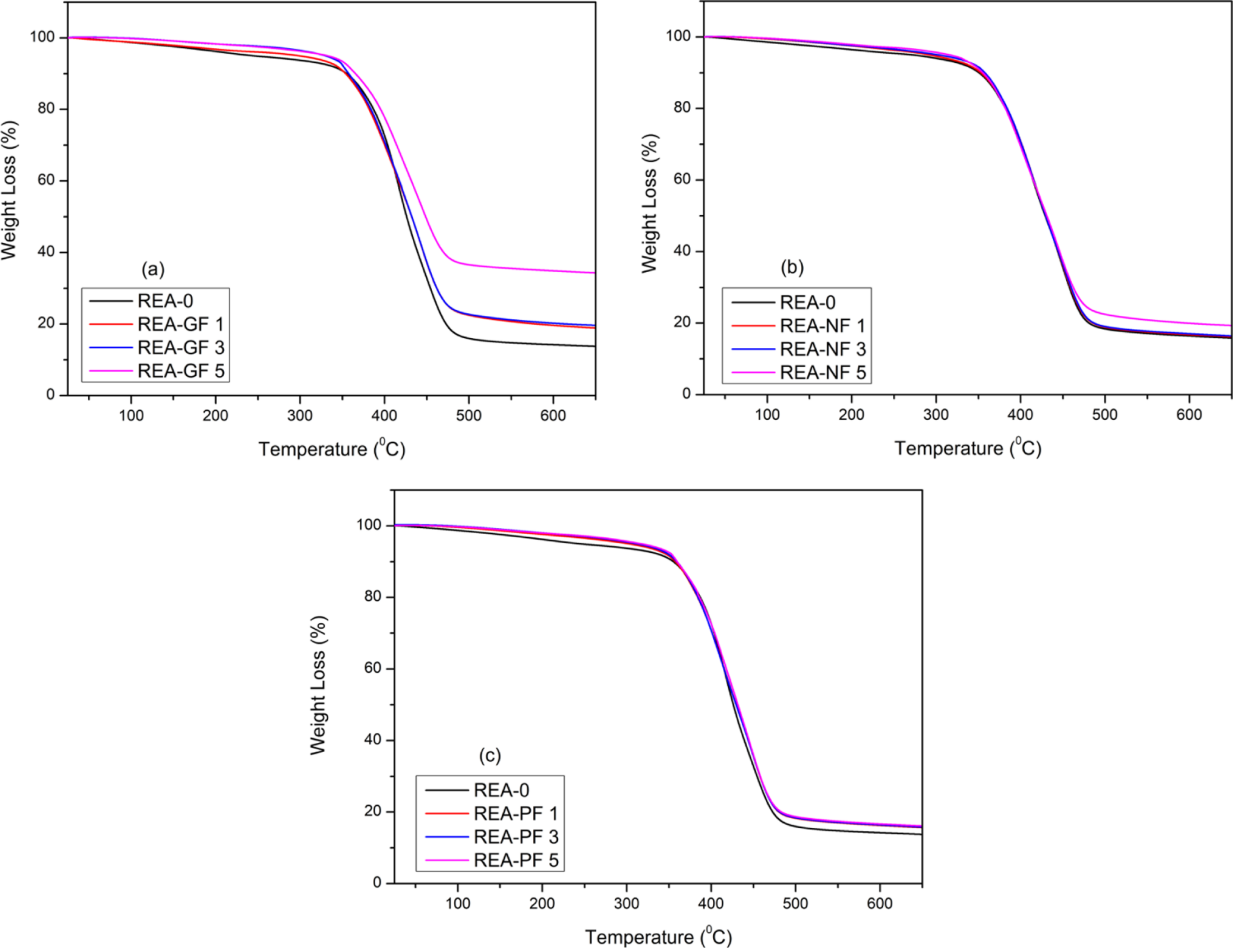
Comparative TGA of UV-cured reinforced composites: (a) REA-GF, (b) REA-NF, and (c) REA-PF with UV-cured non-reinforced REA-0.

As demonstrated in Figure 10, the thermogravimetric (TG) curve indicates one-step degradation for REA-0 and its fiber-reinforced composites. The results elucidate that all the UV-cured composites follow the same degradation method, and their maximum degradation occurred in the range of 350–500 °C. Observing that the entire temperature range indicates that thermal stability increases as the fiber reinforcement percentage increases in the composites. The primary reason for this behavior is the fine dispersion of fibers in UV-cured composites, which builds up efficient interfacial interactions within the resin matrix that lead to an increment in thermal properties of UV-cured composites. The char yield at 650 °C was the lowest for REA-0, whereas it was the highest for UV-cured composites of REA-GF, followed by REA-NF and REA-PF; this mainly depends on the type and percentage of fiber reinforced. Hence, from the TG curve, it can be concluded that the thermal properties of UV-cured composites enhances with the increase in percentage reinforcement of fibers. It also indicates that GF enhances thermal properties to the utmost level, followed by PF and NF. TGA data in Table 3 demonstrate temperatures at different weight losses and char yields for all UV-cured composites.

**Table 3:** TG Data of UV-Cured Samples in Nitrogen^a^^,^^b^^,^^c^^,^^d^

resin formulation (wt %)	*T*_10_ (°C)	*T*_50_ (°C)	*T*_max_(°C)	char yield (650 °C)
REA 0	348	426	524	13.76
REA-GF 1	353	430	541	18.89
REA-GF 3	357	432	583	19.63
REA-GF 5	365	448	590	34.29
REA-NF 1	351	428	621	15.66
REA-NF 3	356	428	628	15.82
REA-NF 5	359	431	643	16.10
REA-PF 1	280	347	584	16.19
REA-PF 3	311	353	592	16.40
REA-PF 5	318	355	597	19.33

a *T*_10_ (°C) is the temperature at 10 wt % weight loss.

b *T*_50_ (°C) is the temperature at 50 wt % weight loss.

c *T*_max_ (°C) is the temperature when degradation has reached maximum.

d Char yield (600 °C) is the residue wt % at 600 °C.

### High-Resolution Microscopic Analysis

**Figure 11:**
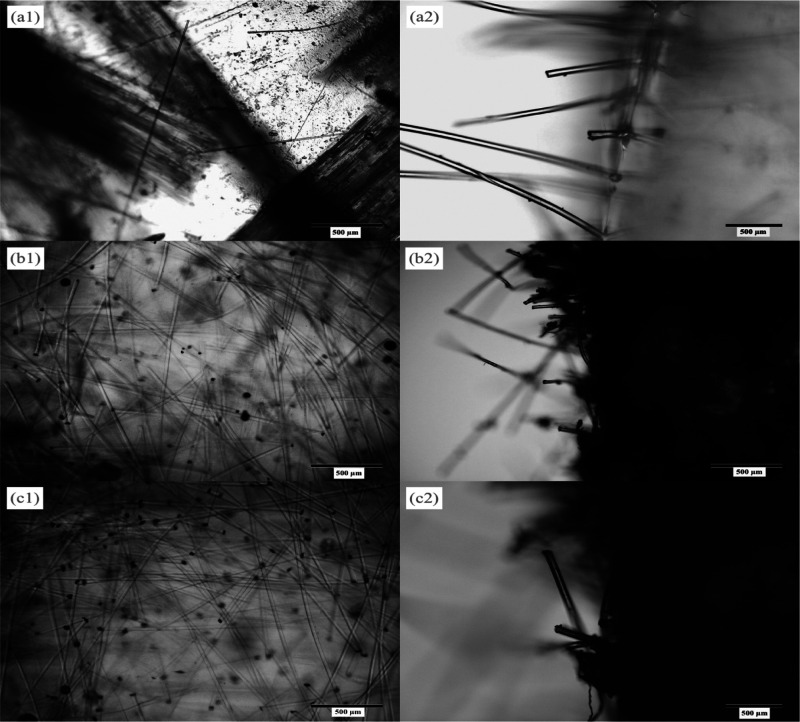
High-resolution microscopic image of UV-cured composites: (a) glass fiber-reinforced composite, (b) nylon fiber-reinforced composite, and (c) polyester fiber-reinforced composite. Image 1 shows the dispersion of fibers, and image 2 shows a cross-sectional view of fiber embedment at a fractured surface.

As demonstrated in Figure 11, different UV-cured composites comprising 3% fiber loading were analyzed using high-resolution microscopic images. We further performed the comparative examination based on the dispersion of fibers and their embedment at the fractured surface. The images conclude that NF produces the best dispersion, followed by PF and least in the case of GF. Looking at the fracture morphology, mechanical interlocking of fibers with the cured resin was the highest in the case of GF, which gives the best inter-surface adhesion, whereas PF exhibits poor adhesion.

## Conclusions

The REA oligomer was synthesized and formulated, conferring to the compatibility of the SLA 3D printer and further dispersing different synthetic fibers in the range of 1, 3, and 5% to formulate fiber-reinforced photocuring resins and create reinforced 3D objects using the SLA technology. The rheological behavior of these fiber-reinforced formulations was studied, and formulations delivering suitable viscous and processability in SLA 3D printers were preferred. Analyzing all the UV-cured composites indicated that NF and PF exhibited excellent stability and homogeneity in the formulated REA resin, whereas incorporating GF causes poor dispersity. However, GF-reinforced composites demonstrated superior physicochemical properties, optical transmittance, mechanical strength, and thermal stability, followed by NF- and PF-reinforced composites. Further, SLA-printed 3D objects crafted from 3% fiber-reinforced formulations demonstrate a good printing resolution and accuracy for all. However, we observed high agglomeration and sedimentation in the case of GF-reinforced printed objects. Thus, looking at all the prospects, NF and PF were considered suitable materials for reinforced SLA 3D printing.
